# Neurological injury in patients with acute myocardial infarction undergoing operative myocardial revascularization within 48 h

**DOI:** 10.1186/s13019-026-04181-y

**Published:** 2026-06-03

**Authors:** Frederik Heumüller, Katharina Huenges, Nourane Trigui, Aysun Tulun, Bjarne Markscheffel, Bernd Panholzer, Tim Attmann, Alexander Thiem, Hanna Gravert, Patrick Langguth, Wiebke Sommer, Gregor Warnecke, Christina Grothusen

**Affiliations:** 1 Department of Cardiac Surgery, University Hospital Schleswig-Holstein, Campus Kiel Arnold-Heller Strasse 3, Haus C, 24105 Kiel, Germany; 2 Institut für Medizinische Informatik und Statistik, Christian-Albrechts-Universität zu Kiel and University Hospital Schleswig-Holstein, Campus Kiel, 24105 Kiel, Germany; 3 Department of Radiology and Neuroradiology, University Hospital Schleswig- Holstein - Campus Kiel, Kiel, Germany; 4 Medizinische Klinik I, St. Johannes Hospital Dortmund, Dortmund, 44137 Germany

**Keywords:** Acute myocardial infarction, CABG, Neurological injury, Complications

## Abstract

**Background:**

Acute myocardial infarction (AMI) is associated with an increased rate of neurological events (NE). AMI patients, who undergo coronary artery bypass graft (CABG) surgery may be at an even greater risk for peri-operative NE, but detailed data is missing.

**Methods:**

We conducted a retrospective, single-center data analysis of 1628 patients that underwent CABG within 48 h after being diagnosed with AMI. Between 01/2001 and 03/2023, 77 patients (4.7%) suffered from a peri-operative NE. This included 66 (4.0%) thromb-embolic strokes and 11 (0.7%) hypoxic brain damages. We compared the outcome between NE patients and those without (w/o) NE. Primary outcome parameters were 30-day mortality and long-term survival. Secondary outcome parameters included post-operative ICU length of stay, transfusion rates and need for renal replacement therapy (RRT).

**Results:**

Median time from AMI diagnosis to CABG was 7.6 h (4.4–16.4 h). Significantly more NE patients were smokers (*n* = 36(46.8%) vs. *n* = 532(34.5%);*p* = 0.04) and presented with a severely reduced left ventricular function pre-operatively (*n* = 15(20.3%) vs. *n* = 161(11.1%);*p* = 0.02). NE patients had undergone CPR pre-operatively more often than patients w/o NE (*n* = 23(29.9%)vs *n* = 168(10.8%);*p* < 0.001). Accordingly, EuroScore II was significantly higher in NE patients compared to patients w/o NE (7.8 (4.2–14.3) vs. 4.9 (2.8–10.2); *p* < 0.001). Intra-operatively, bypass-time proved to be longer in NE patients (117 (94–149) vs. 107 (88–130)minutes; *p* = 0.02). Post-operatively, significantly more NE patients had to stay longer than 48 h in the ICU (*n* = 72 (94.7%) vs. *n* = 866 (55.8%); *p* < 0.001). Neither transfusion rates nor need for RRT differed between the groups. Thirty day mortality was higher in NE patients (*n* = 16 (20.8%) vs. *n* = 165 (10.7%); *p* < 0.01). Pre-operative diagnosis of peripheral artery disease (pad) and need for CPR were identified as independent predictors of 30-day mortality in NE patients. Ten-year survival of NE patients remained impaired compared to patients w/o NE (39% vs. 69%; *p* < 0.001).

**Conclusion:**

AMI Patients undergoing CABG within 48 h are at an increased risk for neurological injuries. In particular, patients with generalized atherosclerosis and those that underwent CPR pre-operatively, seem to represent a vulnerable subgroup. Further studies have to clarify whether individualized peri-operative actions may reduce the stroke rates in this setting.

**Supplementary Information:**

The online version contains supplementary material available at 10.1186/s13019-026-04181-y.

## Introduction

Patients with acute myocardial infarction (AMI) are in an elevated state of inflammation and hypercoagulation [1]. The risk of cerebrovascular events is increased in this setting and associated with a significantly reduced chance of survival [2]. While contemporary studies demonstrated a stroke rate of approximately 1% for patients that undergo CABG or PCI under elective conditions, neurological event rates vary greatly when these procedures are performed in AMI patients [3, 4]. In particular, patients with cardiogenic shock or AMI-related cardiopulmonary resuscitation (CPR) are at high risk for cerebral complications. In this regard, the ECLS-shock trial as well as the DanGer Shock trial reported a stroke rate of around 4% [5, 6]. The proportion of hypoxic brain damages was reported with 12% [5]. Neurological injuries are particularly feared when CABG is performed in AMI patients as the surgical trauma, general anesthesia and use of extracorporeal circulation may further aggravate systemic inflammation and pro-thrombotic processes. However, clinical data on this subject are rare. We here retrospectively analyzed the rate and nature of neurological events in patients that underwent CABG within 48 h after being diagnosed with AMI at our institution.

## Methods

### Data source

The Kieler myocardial infarction registry was started in 2001. It is an ongoing, single-center registry that includes patients with acute myocardial infarction (AMI) that underwent operative myocardial revascularization within 48 h after diagnosed with AMI. Patients that underwent surgery later than 48 h after the AMI diagnosis had been made, were excluded. Patients were also excluded, when combination procedures other than closure of a patent foramen ovale had to be performed. Further details of this registry have been published elsewhere [7]. For this particular analysis, the outcome of patients with a post-operatively detected neurological event (NE, *n* = 77) were compared to those without neurological events (w/o NE, *n* = 1551). The primary outcome of this sub-group comparison was 30-day mortality and long-term survival over 10 years. The secondary outcome parameters included the length of stay in the ICU, need for post-operative renal replacement therapy and transfusion rates. Patients or their surrogate decision maker provided written consent. In addition to 30-day mortality, follow-up data was obtained. Data was collected by contacting the respective patients by mail. In cases, where patients or relatives did not respond, we interrogated their general practitioner. If whereabouts remained still unknown, we contacted the public records office. The study was approved by the institutional review committee. One patient was lost during the follow-up period. The datasets used and analysed during the current study are available from the corresponding author on reasonable request.

### Evaluation of neurological events

All patients demonstrated neurological symptoms within the phase of awakening from general anesthesia after CABG. In accordance with the local standard operating procedures (SOP) these patients were clinically assessed by consultants of the Department of Neurology and the Department of Neurosurgery, if deemed necessary. All patients underwent an emergency CT scan analysis as the primary diagnostic tool. If CT scans were inconclusive or without any pathological findings explaining the symptoms of the respective patient, an MRI scan was added. We performed a focused multimodal magnetic resonance imaging (MRI) protocol. We acquired axial T2-weighted images to obtain an anatomical overview and to assess edema or chronic tissue changes. Axial diffusion-weighted imaging (DWI) was used to identify early ischemic lesions. Axial susceptibility-weighted imaging (SWI) allowed detection of intracranial hemorrhage or thrombotic material. We additionally obtained axial time-of-flight (TOF) MR-angiography to evaluate intracranial arterial flow and potential vessel occlusions or stenoses. Finally, sagittal fluid-attenuated inversion recovery (FLAIR) imaging was performed to visualize subacute or chronic ischemic changes, periventricular abnormalities as well as potential subarachnoid pathology. All aspects of NE patient care were done in accordance with the recommendations of the Department of Neurology and/or Department of Neurosurgery. A detailed list of the neurological symptoms of the patients included in this analysis is provided in supplemental Table 1.

### Pre-operative management

Every case and time-point of CABG was discussed with the referring cardiologist. Our department usually operates on AMI patients immediately after transfer from the initial treatment centers independent of hemodynamic stability, symptoms or cardiac enzymes. This approach usually excludes patients that present with a definitely subsided myocardial infarction. CABG was performed under dual platelet therapy regardless of the individual substance used.

### Surgical management

A standard median sternotomy and cardiopulmonary bypass (CPB) with moderate hypothermic (34 °C) cardiac arrest was performed in all but one patient. Myocardial arrest was obtained with cold blood cardioplegic solution applied antegrade via the ascending aorta. In cases with total occluded coronary arteries or incomplete cardiac arrest, antegrade cardioplegia was combined with retrograde administration via the coronary venous sinus. The choice of graft was left at the discretion of the surgeon in charge. If bleeding was not a concern, acetylsalicylic acid administered orally starting on post-operative day one.

### Statistical analysis

All statistical analyses were performed with R version 4.3.2 [8]. Hypothesis tests were two-sided and p values < 0.05 were deemed statistically significant. The primary influence variable of interest was neurological event (NE), categorized into two groups: with an NE and without a neurological event (w/o NE). For the descriptive and univariable analysis, nominal and ordinal variables were described as absolute and relative frequencies and compared between the two groups using Fisher´s exact test. For continuous variables, histograms were inspected to check for normal distribution. All continuous variables showed strong deviations from a normal distribution and were described as median and 25th and 75th percentiles, and compared using the Wilcoxon rank-sum test. The outcome of 30-day mortality was analysed by multiple logistic regression. All parameters with a univariable significant association (*p* ≤ 0.05) with 30-day mortality were included as influence variables in the model, except for Euroscore due to its collinearity with several variables. Model selection in the multiple models was performed by backward selection and a p value threshold of 0.05. The multiple logistic regression was performed three times on different populations. First, two stratified analyses (with neurological event and without neurological event) were performed. Here, all variables were tested in the preliminary univariable analyses for selection of influence variables for the multiple model. The whole dataset (including all patients) was then additionally analysed by multiple logistic regression, with 30-day mortality as the outcome and neurological event group as an additional influence variable.

For long-term survival, a Kaplan-Meier curve stratified by neurological event group was created with the R package survival [9]. The two survival curves were then statistically compared using the log-rank test.

## Results

### Neurological event analysis

Overall, 77 (4.7%) NE occurred peri-operatively (Table 1). A detailed analysis among NE patients revealed that the majority suffered from thromboembolic events (*n* = 66 (85.7%)). Hypoxic brain damages occurred in 11 patients, of which the majority had undergone CPR prior to surgery (9 (81.1%)). Hemorrhagic insults were not detected.

**Table 1:** Detailed analysis of neurological events

Parameter	*N* (%)
Neurological events	77
-Thrombembolic stroke	- 66 (85.7)
-Hypoxic brain damage	- 11 (14.2)
-After CPR	9 (81.8)

CPR: cardiopulmonary resuscitation

### Pre-operative details

NE patients were significantly older (70 years (65–77) vs. 68 (60-74.5); *p* = 0.02) and more frequently active smokers prior to surgery (*n* = 36 (46.8%) vs. *n* = 532 (34.5%); *p* = 0.04). In addition they repeatedly presented with a severely impaired left ventricular function (*n* = 15 (20.3%) vs. *n* = 151 (11.1%); *p* = 0.02), were more likely to have undergone cardio-pulmonary resuscitation (CPR) pre-operatively (*n* = 23 (29.9%) vs. *n* = 168 (10.8%);*p* < 0.001) and were more often on a ventilator prior to surgery (*n* = 18 (23.4%) vs. *n* = 185 (11.9%);*p* = 0.007). As a result, NE patients showed a higher EuroScore II compared to patients w/o NE (7.8 (4.2–14.3) vs. 4.9 (2.8–10.2); *p* < 0.001) (Table 2). As depicted in Tables 3 , 17 (23.3%) NE patients compared to 447 (29.7%) patients w/o NE underwent CABG on dual anti-platelet therapy (DAPT) without differences between the groups (*p* = 0.24). Almost 50% of patients in both groups were diagnosed with coronary left main stenosis (*n* = 38 (49.4%) v *n* = 680 (43.7%); *p* = 0.23) and coronary 3-vessel disease (*n* = 66 (85.7%) vs. *n* = 1291 (83.2%); *p* = 0.42). Duration from AMI diagnosis to start of surgery was between 7 and 8 h (8.5(4.13-12.0) vs. 7.6 (4.4–16.4);*p* = 0.95) with no differences between the groups.

**Table 2:** Patient characteristics

Parameter	w/o NE (*n*=1551)	NE (*n*=77)	*p*-value
Age (years) (median (IQR))	68 (60-74.5) (*n*=1551)	70 (65-77) (*n*=77)	*0.02
Female (n (%))	355/1551 (23.0)	17/77 (22.1)	1.00
BMI [kg/m^2^] (median(IQR))	27.0 (24.7-30.1) (*n*=1549)	27.2 (24.3-31.0) (*n*=77)	0.95
Diabetes (NIDDM) (n (%))	176/1551 (11.3)	8/77 (10.3)	0.50
aHT (n (%))	1182/1550 (76.3)	58/77 (75.3)	0.89
HLP (n (%))	702/1549 (45.3)	29/77 (38.0)	0.20
Smoking (n (%))	532/1542 (34.5)	36/77 (46.8)	*0.04
PAD (n (%))	178/1551 (11.5)	15/77 (19.5)	0.05
RRT (n (%))	16/1549 (1.0)	2/76 (2.5)	0.20
Atrial fibrillation (n (%))	162/1549 (10.5)	13/77 (17.0)	0.09
LVEF<30% (n (%))	161/1455 (11.1)	15/77 (20.3)	*0.02
Prior cardiac surgery (n (%))	15/1550 (1.0)	2/77 (2.6)	0.09
STEMI (n(%))	625/1546 (40.3)	29/77 (37.7)	0.72
NSTEMI (n (%))	920/1546 (59.3)	47/75 (61.0)	0.63
Stroke (n (%))	97/1551 (6.3)	9/77 (11.7)	0.09
EuroScore II (n (%))	4.9 (2.8-10.2) (*n*=1442)	7.8 (4.2-14.3) (*n*=67)	*<0.001
Cardiogenic shock (n (%))	250/1550 (16.1)	16/77 (20.8)	0.27
Intubated (n (%))	185/1550 (11.9)	18/77 (23.4)	*0.007
CPR (n (%))	168/1551 (10.8)	23/77 (29.9)	*<0.001

NE: neurological event; NIDDM: non-Insulin-dependent diabetes mellitus; BMI: body mass index; aHT: arterial hypertension; HLP: hyperlipidemia; PAD: peripheral arterial disease; RRT: renal replacement therapy; LVEF: left ventricular ejection fraction; CPR: cardio-pulmonary resuscitation; STEMI: ST-elevation myocardial infarction; Values are mean(±SD), n (%) or median (interquartile range (IQR)) as indicated. P-values <0.05 were considered as statistically significant

**Table 3:** Coronary anatomy and interventions prior to surgery

Parameter	w/o NE(n=1551)	NE(N=77)	p-value
No oral anti-platelet treatment	701 (45.1)	38 (49.3)	0.48
Intravenous ASA prior to surgery	1551 (100)	77 (100)	1.00
DAPT (n(%))	447 (28.8)	17 (22.0)	0.24
ASA+clopidogrel (n (%))	327 (21.0)	12 (15.5)	0.31
ASA+Prasugrel (n (%))	13 (0.8)	0 (0.0)	1.00
ASA+Ticagrelor (n (%))	107 (6.8)	5 (6.4)	1.00
Coronary left main stenosis (n (%))	680 (43.8)	38 (49.3)	0.34
1-VD (n (%))	45 (2.9)	2 (2.5)	1.00
2-VD (n (%))	211 (13.6)	7 (9.0)	0.30
3-VD (n (%))	1296 (83.5)	67 (87.0)	0.52
PCI<48hrs (n (%))	276 (17.7)	11 (14.2)	0.53
PCI failure (n (%))	116 (42.0)	9 (81.8)	0.18
PCI complication (n (%))	71 (25.7)	1 (9.0)	0.25
In-Stent thrombosis (n (%))	12 (4.3)	0	1.00
Incomplete revascularization with ongoing ischemia (n (%))	24 (8.6)	1	1.00
Large thrombus (n (%))	7 (2.5)	0	1.00
Unknown (n (%))	46 (16.6)	0	1.00
PCI culprit lesion (n (%))	272 (17.5)	12 (15.5)	0.75
Lysis (n (%))	71 (4.5)	7 (9.0)	0.09
Lactate >2mmol/l (n (%))	271 (17.4)	13 (16.8)	1.00
Time from diagnosis to surgery(hours; median (IQR))	7.6 (4.4-16.4)	8.5 (4.13-12.0)	0.96

NE: neurological event; DAPT: dual anti-platelet therapy; ASA: acetylsalicylic acid; VD: vessel disease; PCI: percutaneous coronary intervention; Values are mean (±SD), n (%) or median (interquartile range (IQR)) as indicated. P-values <0.05 were considered as statistically significant

**Table 4:** Intra-operative details

Parameter	w/o NE (*n* = 1551)	NE (*n* = 77)	*p*-value
Procedure time (min) (median (IQR))	220 (190–255) (*n* = 1544)	230 (195–270) (*n* = 77)	0.05
Bypass time (min) (median (IQR))	107 (88–130) (*n* = 1538)	117 (94–149) (*n* = 77)	*0.02
Cross-clamp time (min) (median (IQR))	64 (50–79) (*n* = 1545)	70 (54–85) (*n* = 75)	0.06
No of dist. Anastomoses (median (IQR))	3 (3–4) (*n* = 1551)	3 (3–4) (*n* = 77)	0.06
Complete revascularization (n (%))	1259/1450 (86.8)	57/64 (89.1)	0.71
LITA+venous grafts (n (%))	1188/1551 (76.6)	66/77 (85.7)	0.07
TAR (n (%))	138/1413 (8.9)	5/77 (6.5)	0.70
Closure of PFO	138/1551 (8.9)	1/76 (1.3)	*0.01

NE: neurological event; min: minutes; LITA: left internal thoracic artery; TAR: total arterial revascularization; PFO: persistent foramen ovale; mean (± SD), n (%) or median (interquartile range (IQR)) as indicated. P-values < 0.05 were considered statistically significant

**Table 5:** post-operative details

Parameter	w/o NE (*n* = 1551)	NE (*n* = 77)	*p*-value
Revision due to bleeding (n (%))	110/1539 (7.2)	9/77 (11.7)	0.17
ICU>48 h (n (%))	866/1529 (55.8)	72/76 (94.7)	*<0.001
> 3 RBC units (n (%))	637/1523 (41.8)	38/75 (50.7)	0.15
New need for RRT (n (%))	186/1531 (12.2)	14/73 (19.2)	0.10
New atrial fibrillation (n (%))	330/1534 (21.5)	17/77 (22.1)	0.90
ECLS (n (%))	51/1548 (3.3)	4/75 (5.3)	0.32
impaired wound healing (n (%))	21/1536 (1.4)	3/74 (4.1)	0.10
30-day mortality (n (%))	165/1537 (10.7)	16/77 (20.8)	*0.01

NE: neurological events; ECLS: extracorporeal life support; RRT: renal replacement therapy. Values are mean (± SD) or n (%) as indicated. P-values < 0.05 were considered as statistically significant

### Intra-operative details

As depicted in Table 4, there was a tendency towards longer procedure times in patients suffering from neurological events post-operatively (230 min (195–270) vs. 220 (190–255); *p* = 0.05) mainly caused by a significantly longer bypass time in this group (117 min (94–149) vs. 107 (88–130); *p* = 0.02). No differences in the median number of distal anastomoses (3 (3–4) vs. 3 (3–4); *p* = 0.06) or complete revascularization (*n* = 57 (89.1%) vs. *n* = 1259 (86.8%);*p* = 0.71) was observed. Use of the left internal thoracic artery (LITA) as bypass graft was high (*n* = 66 (85.7%) vs. *n* = 1188 (76.6%);*p* = 0.07) but total arterial revascularization rates were low (*n* = 5 (6.5%) vs. *n* = 138 (8.9%);*p* = 0.70).

### Post-operative details

NE patients endured a longer stay in the ICU compared to those without neurological injuries (*n* = 72 (94.7%) vs. *n* = 866 (55.8); *p* < 0.001) (see Table 5). No differences between need for revision surgery due to bleeding (*n* = 9 (11.7%) vs. *n* = 110 (7.2%); *p* = 0.17) or new need for renal replacement therapy (*n* = 14 (19.2%) vs. *n* = 186 (12.2%); *p* = 0.10) were observed. Thirty day mortality was significantly higher in patients with a neurological event (*n* = 16 (20.8%) vs. *n* = 165 (10.7%); *p* = 0.01).

### Risk factors for 30-day mortality

As shown in Table 6, pre-existing peripheral artery disease (pAD) and cardio-pulmonary resuscitation (CPR) prior to surgery were identified as independent risk factors for 30-day mortality in NE patients.

**Table 6:** Predictors for 30-day mortality

Patients with NE			
Parameter	OR	CI	*p*-value
pAD	7.244	1.778 - 29.508	0.0057
CPR prior to surgery	5.910	1.593–21.928	0.0079

Patients w/o NE			
Parameter	OR	CI	p-value
pAD	2.549	1.490–4.359	0.0006
LVEF < 30%	2.428	1.455–4.051	0.0007
Intubated	1.912	1.035–3.533	0.0384
Pre-op Lactate>2mmol/l	3.254	2.058–5.144	< 0.001
Cardiogenic shock	3.535	1.979–6.316	< 0.001
Age (years)	1.064	1.037–1.091	< 0.001
Left main stenosis	1.617	1.057–2.473	0.0267

In patients w/o NE, 30-day survival was particularly threatened in patients with pre-operatively elevated lactate levels >2mmol/l and individuals with cardiogenic shock.

### Long-term survival

**Fig. 1:**
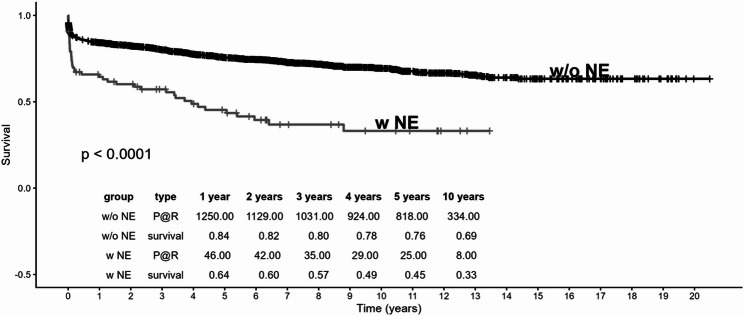
10-year survival of patients with neurological event (NE) versus without a neurological event (w/o NE). Kaplan-Meier curve demonstrating a significantly reduced survival of NE patients over 10 years. P@R: patients at risk

As shown in Fig. 1, Kaplan-Meier analysis revealed that long-term survival over 10 years remained significantly impaired in NE patients (39% survival rate in NE patients vs. 69% survival rate in patients w/o NE; *p* < 0.001) .

## Discussion

Neurological events are among the most feared complications in context with cardiac surgery. Under elective conditions, stroke rates range between under one and around 3%, depending on the selection of patients as well as severity of coronary artery disease (CAD)[ 3, 4].

Contemporary randomized data for neurological events among AMI patients exclusively exists for patients undergoing PCI [10, 11]. These data show, that the likelihood for any neurological injury seems to be approximately 10 times higher than under elective conditions. Cardiogenic shock or CPR, however excessively increase the risk for neurological damage – either thromboembolic or hypoxic [5, 6]. No comparable data exists for patients with AMI referred to CABG, particularly, for patients who undergo CABG with ongoing myocardial ischemia [12, 13]. Our data show an overall rate of cerebrovascular events of 4.7%. The vast majority of these complications were categorized as thromboembolic while hypoxic brain damages were mostly found in patients that had undergone pre-operative CPR. The need for CPR- along with pAD was also identified as an independent risk factor for 30-day mortality in NE patients. In a recent analysis among 1631 patients, of which 124 underwent CABG within 24 h after being diagnosed with AMI, stroke rate was 4.8%. However, patients who had undergone CPR prior to surgery had been excluded [14]. Our institution offers operative revascularization for all AMI patients. Thus, our data includes cases, where PCI was unsuccessful or resulted in complications. In addition, we also offer operative treatment when a complete revascularization strategy appears to be the more sustainable approach upfront. These decisions are made in interdisciplinary discussions with the referring cardiologists and are based on the indications for interventional treatment as recommended in current guidelines [12, 13]. We are of the opinion that– in accordance with accumulating data from interventional trials and recent guideline recommendations – that the fear of complications should not exceed the potential, long-term survival benefit of complete, timely revascularization, especially when CAD is complex. Randomized trials covering the time point of operative revascularization in AMI patients are not likely to be carried out anytime in the near future. Therefore, registry data remain the only way to investigate this topic. Our data show, that neurological event rates are increased, when CABG is performed timely in AMI. Despite these manageable likelihoods, the outcome of AMI patients with neurological injuries remains a matter of concern. In this regard, our analyses demonstrate an increase in length of stay in the ICU as well as a significantly impaired short- and long-term mortality. These results are in line with other reports covering the outcome after cerebrovascular events in AMI patients treated with PCI or CABG [15, 16].

Therefore, identification of patients at increased risk for neurological events may help to discuss treatment strategies and optimize peri-operative management. In addition to the role of pre-operative CPR, our data also demonstrated that NE patients were older, more often active smokers and frequently presented with severe LV-dysfunction. These characteristics are also known to increase the likelihood for peri-operative strokes after CABG in elective settings [17, 18]. Although peri-operative atrial fibrillation is also an established risk factor for cerebral thromboembolic events in this context, we did not find any differences between patients with and without neurological events regarding this parameter [19]. However, we cannot rule out, that this may either be due to the low number of strokes and/or the fact, that the nature of this study may have resulted in an incomplete documentation of parameters. Full body CT scans performed directly after coronary angiography may present another, readily available tool to further facilitate individual case-based discussions about the optimal treatment strategy in AMI patients with complex CAD. While the role of CT imaging has been recognized after successful CPR, a wider range of AMI patients could benefit from this approach. In particular, recognition of atherosclerosis in the ascending aorta as well as carotid stenoses could result in off-pump surgery or a hybrid procedure. Although scientific evidence is scarce, particularly elderly, otherwise hemodynamically stable AMI patients could benefit from these considerations [20]. In addition, CT scans are helpful to identify signs of cerebral damage and other pathologies that may further influence peri-operative neurological risk evaluation and interdisciplinary discussion. In addition, intra-operative neuromonitoring may facilitate recognizing cerebral low-flow and improve the hemodynamic management of AMI patients [21, 22].

## Conclusion

Neurological injury remains one of the most feared complications associated with cardiac surgery in acute myocardial infarction. Our data show that the risk for stroke or hypoxic brain damage is elevated in this setting. Critical, individual case-based heart team discussions are necessary to achieve complete myocardial revascularization.

### Limitations

This study is a single-center, retrospective analysis that covers a very long time period. Thus, the data is prone to selection bias on many levels. In addition, treatment approaches have changed over time and may have influenced the outcome of the respective patients. We did not routinely measure myocardial biomarkers such as high-sensitive troponins. These biomarkers, however may help to identify patients at high risk for post-operative cardiovascular complications [23].

### Future perspective

During recent years, the importance of complete myocardial revascularization in AMI patients has become clear and is supported by current guideline recommendations [12]. CABG has a proven survival benefit for patients with complex CAD under elective conditions. In addition, operative myocardial revascularization has been demonstrated to be superior in AMI patients presenting with cardiogenic shock compared to a medical treatment alone [12]. However, the role of CABG for AMI patients remains unclear [12]. Thus, efforts have to be intensified to investigate the role for a surgical approach embedded in a modern, interdisciplinary peri-operative management. A large randomized study comparing PCI with CABG in AMI patients with complex CAD could clarify the role of operative myocardial revascularization in this setting.

## Electronic Supplementary Material

Below is the link to the electronic supplementary material.

Supplementary Material 1.

## Data Availability

The datasets used and analysed during the current study are available from the corresponding author on reasonable request.
