# Supplementary material for: Neurological injury in patients with acute myocardial infarction undergoing operative myocardial revascularization within 48 h
Source: J Cardiothorac Surg. 2026 Jun 3;21:334. doi: 10.1186/s13019-026-04181-y (PMC13237997; doi:10.1186/s13019-026-04181-y)
Supplement: Supplementary file 1 — Supplementary Material 1. [file 13019_2026_4181_MOESM1_ESM.docx]

| **Clinical symptoms** | **Number of cases** |
| --- | --- |
| hemiparesis | 13 |
| Inadequate awakening, an-isocoria | 4 |
| Inadequate, prolonged awakening | 10 |
| Hemiplegia, apraxia, dysphagia | 5 |
| Inadequate awakening | 9 |
| Awake but unable to contact | 4 |
| sensoric aphasia | 1 |
| Hemiparesis, aphasia | 2 |
| Agitated, hemiparesis | 2 |
| tetraparesis | 1 |
| Visual impairment | 3 |
| Inadequate awakening, hemiparesis | 9 |
| Prolonged awakening, general seizure | 3 |
| Hemiparesis, visual impairment | 2 |
| Myoclonic seizure right arm | 1 |
| Prolonged awakening, general seizure, hemiparesis | 2 |
| aphasia | 1 |
| Inadequate awakening, hemiparesis, visual impairment | 1 |
| Inadequate awakening, positive babinsky reflex | 1 |
| Dysarthria, aphasia, dysphagia, inadequate awakening | 1 |
| Inadequate awakening, hemiparesis, headache | 1 |
| Hemiparesis, dysarthria | 1 |
